# Trk Signaling Inhibition Reduces cSCC Growth and Invasion in In Vitro and Zebrafish Models and Enhances Photodynamic Therapy Outcome

**DOI:** 10.3390/ijms262110434

**Published:** 2025-10-27

**Authors:** Marika Quadri, Natascia Tiso, Marco Iuliano, Paolo Rosa, Roberta Lotti, Giorgio Mangino, Alessandra Marconi, Elisabetta Palazzo

**Affiliations:** 1 DermoLab, Department of Surgical, Medical, Dental and Morphological Sciences, University of Modena and Reggio Emilia, 41125 Modena, Italy; marika.quadri@unimore.it (M.Q.); roberta.lotti@unimore.it (R.L.); alessandra.marconi@unimore.it (A.M.); 2 Department of Biology, University of Padova, 35131 Padova, Italy; natascia.tiso@unipd.it; 3 Department of Medico-Surgical Sciences and Biotechnologies, Sapienza University of Rome-Polo Pontino, 04100 Latina, Italy; marco.iuliano@uniroma1.it (M.I.); p.rosa@uniroma1.it (P.R.); giorgio.mangino@uniroma1.it (G.M.); 4 Istituto Chirurgico Ortopedico Traumatologico (ICOT), 04100 Latina, Italy

**Keywords:** cutaneous squamous cell carcinoma (cSCC), spheroids, zebrafish, Trk signaling, photodynamic therapy (PDT), chemotherapy

## Abstract

Cutaneous squamous cell carcinoma (cSCC) is the second most common skin cancer, with a rising global incidence. Neurotrophins (NTs) and their receptors, including TrkA and CD271, play key roles in epidermal homeostasis and tumor progression. We showed that CD271 expression and function are critical for low- to high-risk progression of cSCC, while TrkA is highly expressed in poorly differentiated tumors. Although NTRK fusions are recognized as oncogenic drivers, the functional impact of TrkA signaling in cSCC remains underexplored. In this study, we investigated the effects of TrkA inhibition, using both the pan-Trk inhibitor K252a and siRNA-mediated silencing, on cSCC cell lines. We evaluated cell growth and invasion in vitro, using 2D and 3D cultures, and in vivo using zebrafish xenografts. TrkA inhibition significantly reduced tumor growth and invasion, with efficacy comparable to standard chemotherapeutics (5-FU, cisplatin). Additionally, TrkA blockade downregulated mitogenic and invasive markers. Importantly, TrkA inhibition enhanced the response to photodynamic therapy in cSCC spheroids. In zebrafish, Trk-targeted interventions reduced metastatic dissemination. These findings highlight TrkA as a key regulator of cSCC survival and metastasis, suggesting its potential as a therapeutic target either alone or in combination with existing treatments.

## 1. Introduction

Cutaneous Squamous Cell Carcinoma (cSCC) represents the second most frequent type of skin cancer, showing a rapid increasing incidence in Europe and the USA by up to 200% in the past 30 years [1,2]. Furthermore, a significant increase in the global burden of cSCC has been observed in recent decades [3,4,5,6,7]. While most SCCs can be easily and successfully treated, if allowed to grow, these lesions can become disfiguring, dangerous, and even deadly. Untreated SCCs can become invasive, grow into deeper layers of skin, and spread to other parts of the body. Due to the high incidence rates, it has been estimated that cSCC accounts for similar death rates as various other cancers, including melanoma and leukemia [8]. Immunocompromised patients have a 65- to 250-fold increased risk of developing cSCC, which is believed to behave more aggressively with a higher metastasis risk [9]. In addition, considering that cSCC typically arises in the latter decades of life, the global increase in life expectancy places its treatment among the key priorities in public health strategies [10]. For low-risk cSCC, different therapeutic options are available, with surgical excision remaining the most common and effective approach [11]. However, in advanced-stage diseases, where surgery may no longer be feasible, radiotherapy and systemic chemotherapy represent alternative strategies. More recently, immune checkpoint inhibitors have emerged as first-line treatments for inoperable or metastatic cases [11,12].

cSCC originates from alterations starting in Keratinocyte Stem Cells (KSCs) and affects their commitment to the maintenance of the epidermal homeostasis. In this context, neurotrophins (NTs) and their receptors form a complex network with regulatory functions within epidermal homeostasis [13]. NTs’ activities are mediated by two classes of receptors, the common NT receptor CD271 and the tyrosine kinase family of receptors (Trks). CD271 binds all NTs with equal low affinity; Trks preferentially interact with a specific NT. In healthy human epidermis, Nerve Growth Factor (NGF) is mainly expressed in KSC and stimulates cell proliferation and survival through the TrkA receptor, thus contributing to the maintenance of “stemness” [13,14].

Specifically, TrkA, encoded by NTRK1, regulates critical cellular functions such as proliferation, survival, and differentiation through its interaction with NGF [15]. Dysregulated Trk signaling has been associated with the development of numerous cancer types, including various solid tumors, such as thyroid, breast, and lung cancers [16]. Among these, gene fusions involving NTRK are the most thoroughly validated oncogenic alterations identified so far [17,18,19]. In both preclinical and clinical contexts, these alterations have emerged not only as key drivers of tumorigenesis but also as potential predictive biomarkers for response to targeted therapies. This has led to the development of a novel class of NTRK inhibitors, some of which have already entered early-phase clinical trials [20,21]. In fact, NTRK fusions are among the most validated molecular alterations in precision oncology and are now targetable with TRK inhibitors such as larotrectinib and entrectinib [15,22].

Beyond gene fusions, functional TrkA signaling has been shown to promote tumorigenicity, angiogenesis, metastasis, and resistance to chemotherapy [23,24,25]. For example, TrkA activity was found to mediate cisplatin resistance in head and neck cancers via the Akt pathway, and its inhibition enhanced the response to immunotherapy in melanoma [26]. Importantly, TrkA and its co-receptors (the common neurotrophin receptor CD271 and sortilin) are expressed in both normal and malignant tissues, with a functional role in tumor progression [27,28].

Recently, Trk signaling has emerged as a potential factor regulating tumor features in terms of proliferation and differentiation in cSCC [13,29]. By combining molecular and histochemical evaluation, we recently demonstrated a direct correlation between TrkA expression and the progression of cSCC in vivo using cSCC patient-derived 3D models [29]. Here, we found that the functional activation of CD271, independently of Trk receptor modulation, had a role in preventing high-risk cSCC tumor development. In this context, after increasing CD271 levels, either by overexpression or ligand-mediated activation, TrkA expression was substantially downregulated, as well as the levels of markers associated with tumor aggressiveness and invasion [29].

In the present study, we evaluated the effects of TrkA inhibition, both pharmacologically via the pan-Trk inhibitor K252a and through siRNA silencing, on the growth, survival, and metastatic behavior of cSCC cells in vitro using 3D models and in vivo using the zebrafish model. Additionally, we explored whether TrkA inhibition can enhance the efficacy of photodynamic therapy (PDT), an established treatment for superficial skin lesions.

## 2. Results

### 2.1. TrkA Inhibition Reduces the Cell Growth and Viability of cSCC Cells

Given the limited data available on the role of Trk receptors in cSCC, we investigated their functional significance in this tumor type. Our previous work highlighted the heterogeneous expression of Trk receptor mRNA in squamous tumors through various omics-based studies [30]. Although a direct functional role for TrkA in cSCC progression has not been fully clarified at the molecular level, it is reasonable to hypothesize its involvement based on the established connection between hyperproliferation and tumor stage. Our recent findings suggest that disrupting neurotrophin signaling, achieved through Trk chimera, can synergize with the increased activation of CD271 to influence tumor dynamics [29]. Collectively, these observations support the idea of exploring TrkA as a potential regulator of cSCC cell behavior.

**Figure 1:**
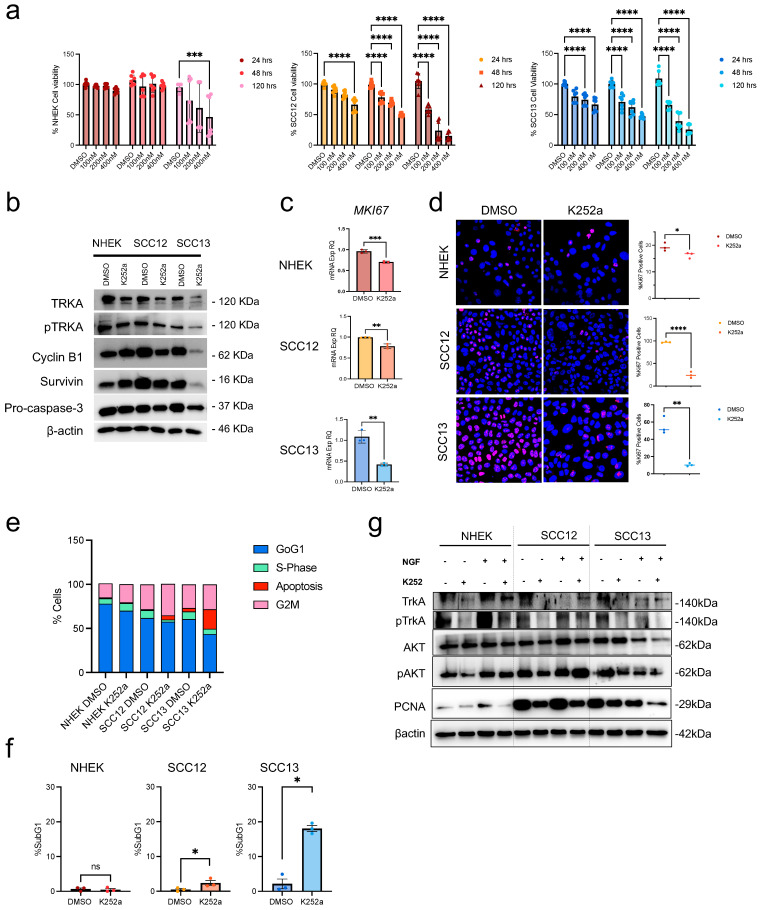
Trk signaling inhibition by K252a affects cSCC growth and viability. (**a**) Cell viability evaluated in NHK, SCC12 and SCC13 cells after treatment with K252a (100, 200 or 400 nM) at 24, 48 and 120 h by MTT assay. (**b**) TrkA, P-TrkA, Cyclin B1, Survivin, and Pro-caspase-3 expression evaluated by Western blotting at 24 h after K252a (200 nM) treatment; b-actin was used as the loading control. (**c**) Ki-67 mRNA expression evaluated by qPCR; b-actin was used as the housekeeping gene. (**d**) Ki-67 protein expression evaluated by immunofluorescence. Images were taken at 40× magnification. (**e**) FACS analysis and (**f**) % of SubG1 cells. (**g**) TrkA, P-TrkA, AKT, and PCNA expression evaluated by Western blotting at 24 h after K252a (200 nM) treatment, with or without NGF (100 ng/mL); b-actin was used as the loading control. For all experiments, statistical analysis was performed using two-way ANOVA. The results are represented as mean ± SD. *p*-values are indicated as the following *: 0.01 < *p* < 0.05; **: 0.001 < *p* < 0.01; *** 0.0001 < *p* < 0.001; **** *p* < 0.0001.

We treated cSCC cell lines (SCC12 and SCC13) as well as Normal Human Epidermal Keratinocytes (NHEK) with the pan-Trk tyrosine kinase inhibitor K252a, which selectively blocks the kinase activity of the Trk family of neurotrophin receptors (TrkA, TrkB, and TrkC), with the highest affinity for TrkA [31]. We confirmed an earlier dose-dependent decrease in cell viability, starting from 24 h after K252a treatment (100, 200, and 400 nM) in tumor cells, both in SCC12 and SCC13 cells, compared to healthy keratinocytes (Figure 1a). By examining cell appearance in culture, we observed that after 24 h of treatment, K252a does not significantly affect NHEK morphology but induces changes in the SCC13 and SCC12 tumor cell lines, including loss of cell–cell adhesion and elongation, which might be considered as signs of cellular stress (Supplementary Figure S1a). These findings suggest a major effect of TrkA inhibition on tumor cells as compared to healthy keratinocytes at the same time points.

We confirmed the decrease in both TrkA expression and phosphorylation in all the analyzed cells after treatment with K252a (Figure 1b, Supplementary Figure S1b). As demonstrated for other experimental settings and cell types [32,33,34], inhibiting TrkA phosphorylation through K252a results in a reduction in growth and survival signals, leading to a decrease in tyrosine kinase receptor expression itself. When compared to healthy keratinocytes, K252a-treated cSCC cells displayed a lower level of proliferation and increased apoptosis, as indicated by the decreased expression of cyclin B, survivin, and pro-caspase 3, which indicates caspase 3 activation (Figure 1b). Furthermore, cell growth inhibition was further confirmed by Ki-67 mRNA downregulation in all cell types (Figure 1c). Notably, the expression of survivin was increased in NHEK after Trk inhibition, potentially due to the induction of a cell cycle block in G2M (Figure 1e), and consequent reduction in procaspase 3, as previously demonstrated [35], promoting a decrease in Ki-67-positive cells (Figure 1d). Importantly, a significant increase in the subG1-peak was observed for SCC12 and SCC13 treated cells at 24 h after K252a treatment (Figure 1f), thus confirming the morphological and molecular analysis.

It is well known that the binding of mature NGF to TrkA induces receptor dimerization and phosphorylation, which activate downstream signaling pathways, including MAPK, PLCg and PI3K/AKT [36,37]. NGF-TrkA axis in the SCC of the head and neck region can trigger epithelial–mesenchymal transition (EMT) and confer resistance to EGFR inhibitors like erlotinib, suggesting a role for TrkA in modulating AKT-dependent pathways in these tumors [38]. Therefore, to further dissect TrkA signaling inhibition in terms of downstream effects in our system, we evaluated the activation of the mitogenic pathway by analyzing AKT phosphorylation in response to K252a treatment, with or without the addition of NGF (Figure 1g; Supplementary Figure S1e). The inhibition or induction of TrkA phosphorylation following treatment with K252a or NGF, alone or in combination, was assessed by analyzing TrkA and P-TrkA expression levels. Our data confirms previous observations [32,39,40,41], with the downregulation of P-TrkA by K252a clearly evident in both NHEKs and cSCC cells. Conversely, while NGF-induced upregulation of P-TrkA was readily observed in NHEKs, TrkA or P-TrkA expression detected in cSCC cells was more cell line-dependent, most likely associated with receptor stability and/or internalization in the presence of NGF [32,41]. Moreover, this outcome is likely related to the endogenous production of NGF by cSCC cells, as reported in previous and independent studies [29,42,43]. Nevertheless, K252a retained its inhibitory effect on P-TrkA level even in the presence of NGF stimulation in vitro.

By evaluating AKT and P-AKT expressions, we confirmed that their modulation reflects TrkA signaling. All cell types showed a decrease in AKT activity following TrkA inhibition (Figure 1g and Supplementary Figure S1e). However, except for NHEKs, where NGF stimulation induced an increase in AKT phosphorylation, AKT activation in cSCC cells remained comparable to that of the control, corroborating the previous findings. Nevertheless, the inhibitory effect of K252a on AKT phosphorylation persisted, indicating that TrkA blockade effectively downregulates AKT signaling even in the presence of endogenous NGF activity. These data are also sustained by the modulation of PCNA expression, which follows growth inhibition or induction according to K252a or NGF stimulus, respectively, and the amount of Ki-67 positive cells (Supplementary Figure S1e).

Collectively, these findings indicate that pharmacological inhibition of TrkA signaling through K252a selectively impairs the growth and survival of cSCC cells while exerting minimal effects on normal keratinocytes under the same conditions. The observed decrease in TrkA phosphorylation, together with the reduction in downstream AKT activation, highlights the functional relevance of the TrkA–AKT axis in sustaining tumor cell proliferation, as reported in other tumoral contexts [38,44,45]. Moreover, the lower responsiveness to NGF in cSCC cells, despite preserved TrkA expression, supports the concept of a constitutively active or autocrine TrkA signaling loop that may contribute to tumor maintenance. Overall, these results point to TrkA inhibition as a promising strategy to counteract pro-survival signaling in cSCC.

### 2.2. TrkA Inhibition Impairs 3D Spheroid Growth

Three-dimensional (3D) spheroid models have become a valuable tool in cancer research, offering a more physiologically relevant representation of the tumor microenvironment compared to traditional two-dimensional (2D) cultures [13]. By mimicking critical features such as cell–cell and cell–matrix interactions, oxygen and nutrient gradients, and multicellular architecture, spheroids provide a robust platform for evaluating drug penetration, therapeutic resistance, and invasive potential.

**Figure 2:**
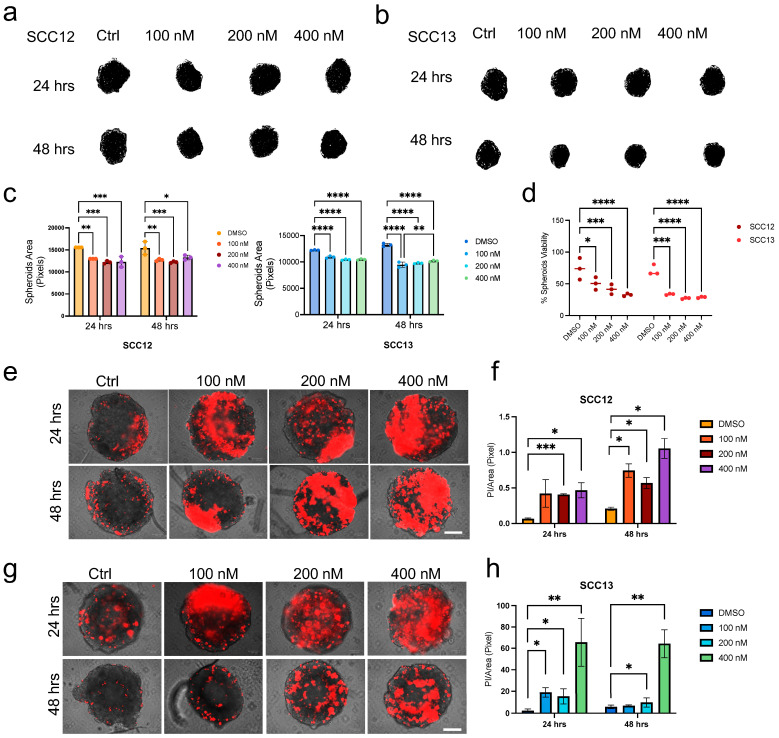
Trk signaling inhibition by K252a decreases growth and invasion in SCC 3D models. (**a**,**b**) Representative picture of SCC12 and SCC13 spheroids after treatment with DMSO (control) or K252a at different doses (100, 200 and 400 nM) at 24 and 48 h. (**c**) SCC12 and SCC13 spheroid, treated as in (**a**,**b**), respectively, total area measured by ImageJ software at 24 and 48 h. (**d**) SCC12 and SCC13 spheroid total area and (**d**) spheroids’ viability evaluated by MTT assay expressed as % with respect to the control at 48 h. (**e**) PI staining/brightfield of SCC12 or (**g**) SCC13 spheroids treated with K252a (100, 200 or 400 nM) or DMSO at 48 h. Scale bar = 100 μm. (**f**) Quantification of SCC12 or (**h**) SCC13 PI staining/Total area ratio. For all experiments, statistical analysis was performed using two-way ANOVA. The results are represented as mean ± SD. *p*-values are indicated as the following *: 0.01 < *p* < 0.05; **: 0.001 < *p* < 0.01; *** 0.0001 < *p* < 0.001; **** *p* < 0.0001.

First, we analyzed the effect of K252a treatment on cSCC-derived spheroids. Image analysis at different time points revealed a significant reduction in area growth rate following treatment (Figure 2a–c). These findings were corroborated by MTT assays (Figure 2d) and PI staining (Figure 2e–h), which confirmed reduced cell viability and increased cell death in both the analyzed cell lines.

These results underscore the critical role of TrkA signaling in sustaining cSCC spheroid growth and invasive behavior, reinforcing the potential of Trk inhibition in disrupting tumor architecture in a 3D context.

### 2.3. Silencing of TrkA Mirrors Pharmacological Inhibition in cSCC

**Figure 3:**
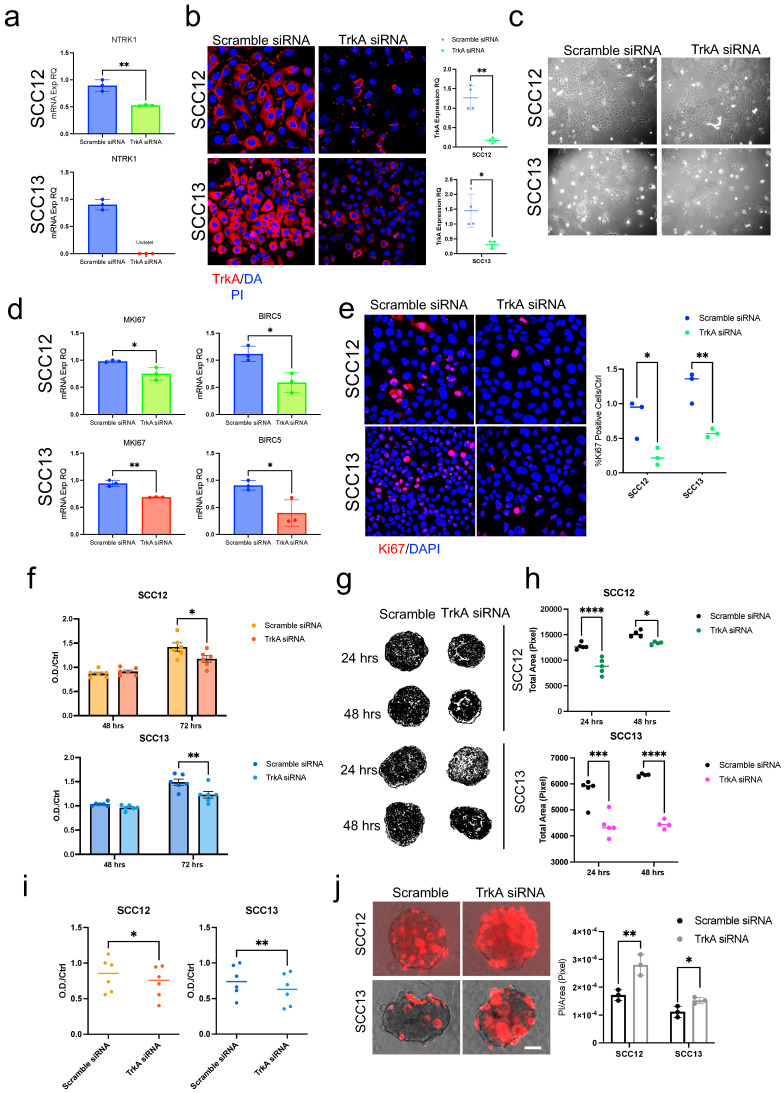
TrkA silencing correlates with lower growth and survival in 2D and 3D models. (**a**) NTRK1 mRNA expression evaluated by qPCR; b-actin was used as the housekeeping gene. (**b**) TrkA protein expression evaluated by Immunofluorescence staining. Images were taken at 40× magnification. (**c**) Representative image of SCC12 and SCC13 cells transfected with Scramble or TrkA siRNA (100 nM). Images were taken at 10× magnification. (**d**) MKI67 and BIRC5 mRNA expressions evaluated by qPCR; b-actin was used as the housekeeping gene. (**e**) Ki67 protein expression level evaluated by Immunofluorescence staining. Images were taken at 40× magnification. (**f**) Scramble or TrkA siRNA-transfected cell viability at 48 and 72 h by MTT assay. (**g**) Representative ImageJ mask of SCC12 and SCC13 Scramble or TrkA siRNA (100 nM) treated spheroids at 24 and 48 h. (**h**) SCC12 and SCC13 Scramble or TrkA siRNA (100 nM) treated spheroid total area measured by ImageJ software at 24 and 48 h. (**i**) SCC12 and SCC13 Scramble or TrkA siRNA (100 nM) treated spheroid viability by MTT assay (**j**) Left panel: PI staining/brightfield of SCC12 and SCC13 spheroids treated with Scramble or TrkA siRNA (100 nM) at 48 h. Scale bar = 50 μm. Right panel: Quantification of SCC12 or SCC13 PI staining/Total area ratio. For all experiments, statistical analysis was performed using two-way ANOVA. The results are represented as mean ± SD. *p*-values are indicated as the following *: 0.01 < *p* < 0.05; **: 0.001 < *p* < 0.01; *** 0.0001 < *p* < 0.001; **** *p* < 0.0001.

Given that K252a has the highest affinity for TrkA, we used siRNA targeting of *NTRK1* to effectively knock down TrkA expression, as confirmed by qPCR and immunofluorescence staining (Figure 3a,b). By TrkA downregulation, cSCC cells did not show an apparent sign of cell death, like those observed for K252a treatment (Figure 3c). However, by proliferation-associated marker analysis, we could confirm a reduced proliferation and cell survival, as indicated by Ki-67 and BIRC5 mRNA downregulation, as well as the number of Ki-67 positive cells (Figure 3d,e).

These results were further confirmed by reduced 2D and 3D cell viability and growth area in both SCC12 and SCC13 cells (Figure 3d–f) following TrkA downregulation. Similar to K252a treatment, PI staining in TrkA-siRNA-treated spheroids indicated increased apoptosis compared to scramble controls (Figure 3f).

These data indicate that the K252a effect is mediated by TrkA receptor signaling inhibition and further support the role in determining the growth and proliferation of the cSCC tumor.

### 2.4. TrkA Inhibition or Silencing Reduces Metastatic Capacity In Vitro

To evaluate if TrkA inhibition influences tumor behavior, also by affecting cell interaction with the 3D tumor environment, we used a more advanced invasion model, where cSCC spheroids were implanted into collagen I matrix in the presence of human dermal fibroblasts.

**Figure 4:**
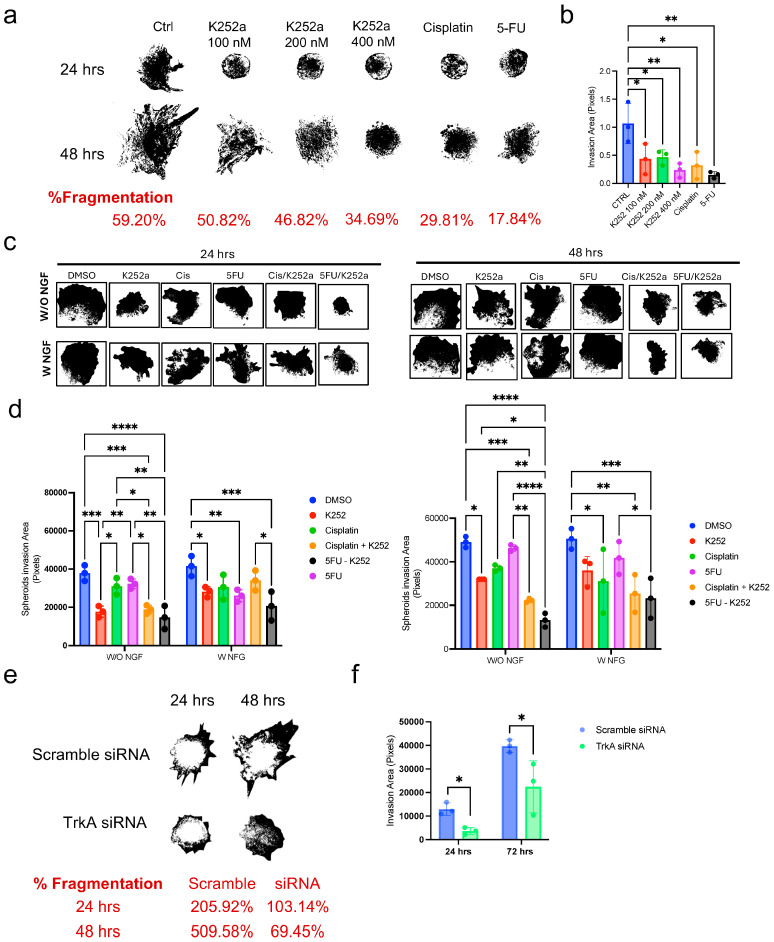
TrkA inhibition or silencing decreases spheroid invasion and improves chemotherapeutic efficacy. (**a**) Upper panel: Representative images of SCC13-derived implanted spheroid treated with K252a, cisplatin or 5-FU monitored for 24 and 48 h. Lower panel: % Fragmentation measured using ImageJ software. (**b**) Total invasion area measured using ImageJ software. (**c**) Representative images of SCC13-derived implanted spheroid treated with K252a, cisplatin or 5-FU or combination, with or without NGF, monitored for 24 and 48 h. (**d**) Total invasion area measured using ImageJ software. (**e**) Upper panel: Representative images of TrkA-silenced SCC13 implanted spheroid monitored for 24 and 48 h. Lower panel: % Fragmentation measured using ImageJ software. (**f**) Total invasion area measured using ImageJ software. For all experiments, statistical analysis was performed using two-way ANOVA. The results are represented as mean ± SD. *p*-values are indicated as the following *: 0.01 < *p* < 0.05; **: 0.001 < *p* < 0.01; *** 0.0001 < *p* < 0.001; **** *p* < 0.0001.

Using different concentrations of K252a, we could observe that all treatments significantly suppressed the invasive spread of the cSCC spheroids and induced a higher degree of spheroid fragmentation, which is linked to cell spreading. Furthermore, even by treating cSCC spheroids with lower K252a doses, these effects were comparable to those observed with conventional chemotherapeutic agents such as cisplatin and 5-Fluorouracil (5-FU) (Figure 4a,b).

Given the efficacy in reducing invasiveness, we also tested whether K252a treatment produced an additive effect with chemotherapeutics cisplatin or 5-FU in reducing cSCC cell spreading. Our data supported the fact that, either in the presence or absence of NGF in the culture media, cSCC spheroid invasive ability is similarly decreased by K252a treatment or its combination with chemotherapeutics (Figure 4c,d; Supplementary Figure S2). Therefore, even in the presence of a TrkA receptor-specific and pro-invasiveness stimulus, the K252a inhibitory effect is still detectable. Specifically, both the combination of Cisplatin or 5-FU with K252a appeared to be more effective in decreasing the invasion area at 24 and 48 h, which means that Trk block can potentially enhance the effect of these chemotherapeutics.

To assess if this effect is specifically dependent on TrkA inhibition, we implanted TrkA siRNA-treated spheroids, as well as Scramble siRNA-treated controls, at 48 h after transduction. Invasion into the collagen matrix was monitored up to 72 h (Figure 4e,f) following implant. Here, we confirm that TrkA silencing affects the invasiveness ability in vitro of the cSCC spheroids. Also, both K252a (Figure 4a) and TrkA silencing produce a reduction in the % of fragmentation.

Therefore, these data support TrkA-specific inhibition as a promising therapeutic strategy, capable of disrupting tumor structure and viability in complex 3D tumor environments.

### 2.5. TrkA Inhibition Reduces Metastatic Capacity In Vivo

The zebrafish (*Danio rerio*) has recently emerged as a new tool for cancer research [13]. We have recently defined for the first time its use in cSCC, by dissecting the role of CD271 signaling in blocking cSCC metastasis [29]. Given that the activation of CD271 correlates with decreased expression of TrkA, we evaluate the metastatic behavior of the cSCC cells after K252a treatment or TrkA silencing.

**Figure 5:**
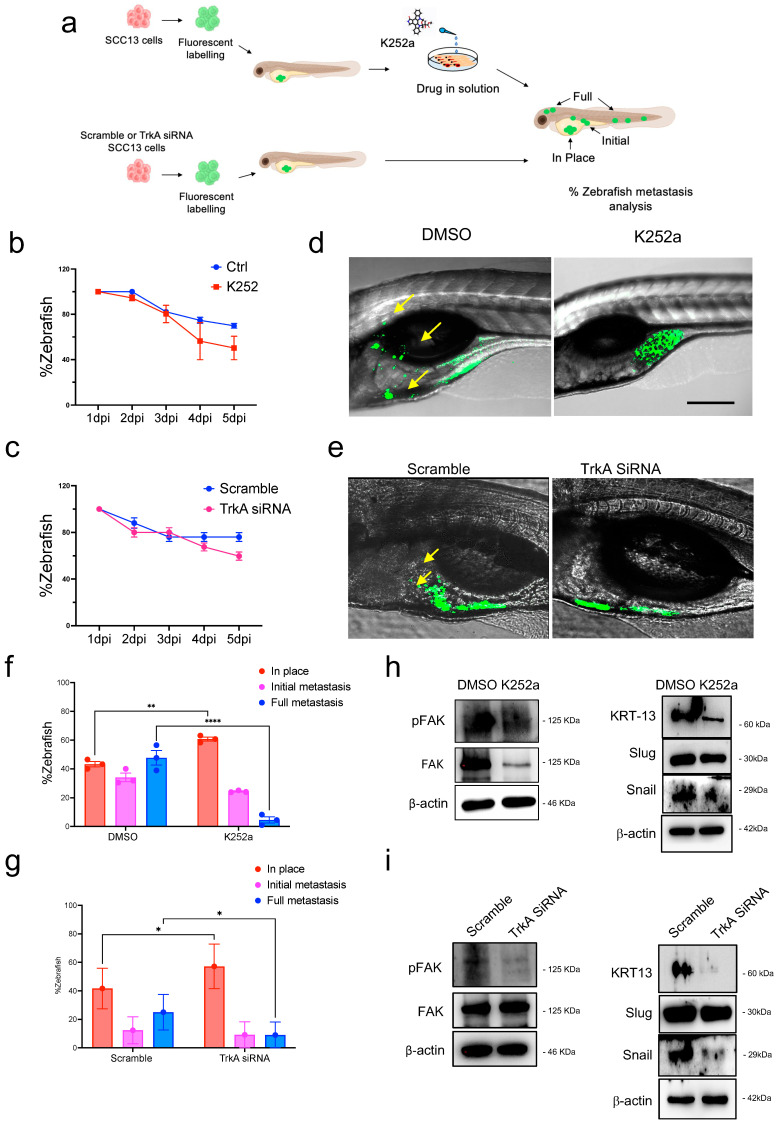
K252a treatment or TrkA silencing decreases the cSCC metastatic capacity. (**a**) Schematic representation of SCC13 cell (green; about 50 cells/embryo) injection into zebrafish larvae according to the experimental condition. (**b**) Zebrafish viability from 0 to 5 dpi, after K252a (200 nM) addition to fishwater or (**c**) for zebrafish injected with scramble or TrkA siRNA-treated SCC13 cells. (**d**) Representative images of zebrafish injected with SCC13 (green) at 5 dpi, after K252a or DMSO (control) treatment, or (**e**) with scramble or TrkA siRNA-treated SCC13 cells (green). Yellow arrows indicate metastatic cells. (**f**,**g**) Metastases were quantified and classified as Full metastases, Initial metastases, and In-place. Results are shown as the mean percentage of three independent experiments and a minimum of 20 injected zebrafish for each condition was used. (**h**,**i**) Let panel: pFAK and FAK expression evaluated by Western Blotting. Right panel: Keratin-13 (KTR13). Slug and Snail expression evaluated by Western Blot. b-actin was used as the loading control. For all experiments, statistical analysis was performed using two-way ANOVA or Student’s *t*-test. *p*-values are indicated as the following *: 0.01 < *p* < 0.05; **: 0.001 < *p* < 0.01; **** *p* < 0.0001.

Fluorescent SCC13 cells have been injected into transparent zebrafish larvae, and cell spreading was evaluated according to the schematic representation in Figure 5a. No differences in fish viability were observed in zebrafish injected with cSCC and treated with K252a at 5 dpi (day post-injection) or injected with TrkA-silenced cells, as compared to control (Figure 5b,c). In both these experiments, Trk signaling inhibition abolishes full metastases and reduces the initial metastases, while increasing the percentage of “in place” cells at 5 dpi (Figure 5d–g).

Furthermore, the expression and activation of Focal Adhesion Kinase (FAK) were assessed by Western blot analysis in cells injected into zebrafish. FAK is a non-receptor tyrosine kinase that plays a critical role in cellular processes such as adhesion, migration, invasion, polarity, proliferation, and survival. Increased FAK expression or activity has been associated with enhanced tumor invasiveness and aggressiveness [46,47,48,49]. In SCC13 cells treated with K252a, both FAK expression and activation were reduced compared to untreated controls (Figure 5h, left panel). Similarly, TrkA-silenced cells exhibited decreased FAK activation relative to scramble-transfected cells (Figure 5i, left panel). cSCC cell phenotype was further assessed by the analysis of KRT13, which reflects aggressive behavior of squamocarcinoma cells [50], and Slug and Snail expression, which are associated with invasion and metastasis [51,52]. Both K252a treatment and, specifically, TrkA silencing affect their expression by reducing their levels (Figure 5h,i, right panel), therefore confirming the decreased ability of cSCC cells to give rise to metastasis in the Zebrafish model.

Altogether, these results strongly support that the downregulation of Trk signaling is instrumental in inhibiting the invasiveness of cSCC cells in vivo and disrupts the metastatic cascade in cSCC by modulating key pro-invasive pathways, including FAK and EMT markers.

### 2.6. TrkA Inhibition Sensitizes cSCC Spheroids to PDT

Photodynamic therapy (PDT) is a minimally invasive procedure that combines the use of a photosensitizing agent, such as Metvix, light of a specific wavelength, and molecular oxygen to generate reactive oxygen species (ROS), leading to targeted tumor cell death. PDT has been successfully applied in the treatment of various superficial malignancies, including actinic keratosis, basal cell carcinoma, and early-stage cSCC. Its advantages include selective cytotoxicity, tissue preservation, and minimal systemic toxicity [53,54].

**Figure 6:**
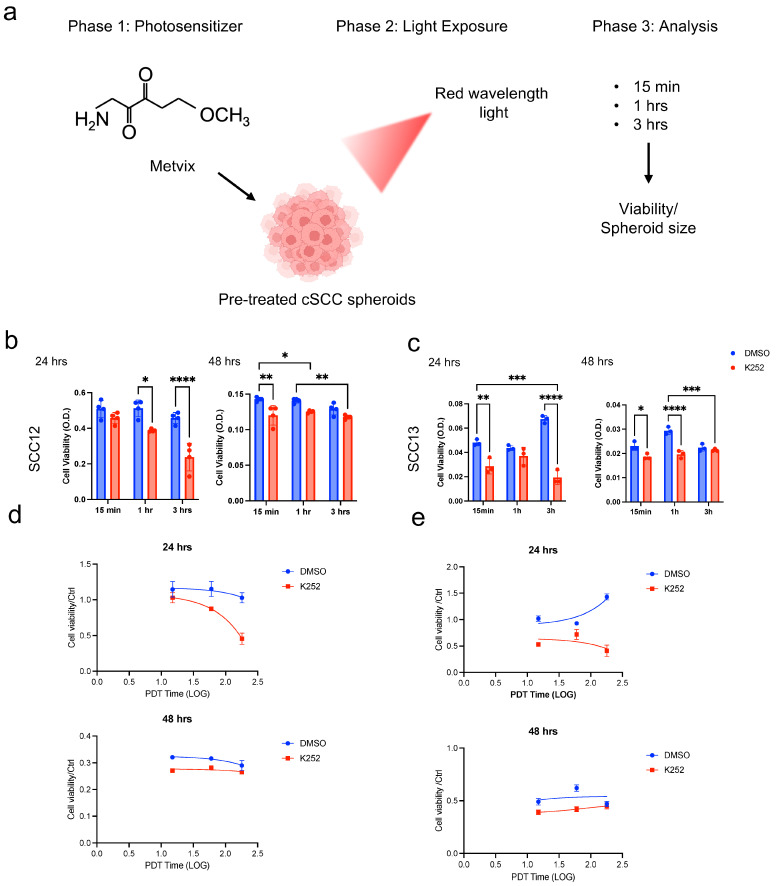
Trk signaling inhibition improves PDT efficiency. (**a**) Schematic representation of photodynamic therapy (PDT) on SCC spheroids. (**b**,**c**) Cell viability evaluated at 24 or 48 h in K252a SCC12 or SCC13-derived spheroids treated with PDT after exposure to Metvix for 15 min, 1 h or 3 h (*n* = 3). Scatter plot XY of the interaction between K252a treatment and Metvix incubation time on SCC12 (**d**) or SCC13 (**e**) spheroid viability by Two-way ANOVA. For all experiments, statistical analysis was performed using two-way ANOVA. The results are represented as mean ± SD. *p*-values are indicated by the following *: 0.01 < *p* < 0.05; **: 0.001 < *p* < 0.01; *** 0.0001 < *p* < 0.001; **** *p* < 0.0001.

As previously reported in Quadri et al. 2023 [29], we exposed cSCC spheroids to Alkilite CL128lamp, after stimulation with Metvix for 15 min, 1 h, and 3 h, to resemble patient treatment. Spheroids’ viability was evaluated at 24 and 48 h. A schematic representation of the treatment is given in Figure 6a. We found that a pre-treatment with K252a enhanced PDT-induced cytotoxicity in both SCC12 and SCC13 spheroids. Spheroid viability were significantly reduced post-PDT, particularly after 3 h from light exposure (Figure 6b,c).

In detail, in SCC12 spheroids, two-way ANOVA revealed a significant interaction between K252 and Metvix time (*F*(2, 12) = 12.24, *p* = 0.0013; ≈15% variance), indicating time-dependent modulation of the K252 effect. Both main effects were highly significant: K252 (*F*(1, 12) = 70.98, *p* < 0.0001; 42% variance) and time (*F*(2, 12) = 29.83, *p* < 0.0001; 36% variance). The graph (Figure 6d, upper panel; Supplementary Table S1) demonstrates a clear reduction in MTT signal after PDT in K252-treated spheroids, particularly when light exposure followed 1 h or 3 h of Metvix incubation, suggesting a strong synergistic enhancement of PDT efficacy. At 48 h, the interaction between K252 and Metvix time was not statistically significant (*F*(2, 12) = 2.77, *p* = 0.10), suggesting that the effect of K252 was comparable across all Metvix times. The corresponding graph (Figure 6d, bottom panel; Supplementary Table S1) always shows consistently lower viability in K252-treated spheroids relative to controls, with no further enhancement over longer incubation periods. This suggests that the maximal sensitizing effect of K252 was already achieved by 24 h.

Similar consideration can be made for SCC13 spheroids. At 24 h, the interaction between the K252 and Metvix incubation time factors was extremely significant (*F*(2, 12) = 52.14, *p* < 0.0001), accounting for ≈ 24% of total variance. This indicates that the effect of K252 on cell viability depended strongly on incubation time. The main effects of K252 (*F*(1, 12) = 303.8, *p* < 0.0001; 70% of total variance) and time (*F*(2, 12) = 6.69, *p* = 0.0112; 3% of variance) were both significant. In the corresponding graph (Figure 6e, upper panel; Supplementary Table S1), K252 pretreatment markedly reduced cell viability, with the maximal decrease observed when PDT was applied 1 h after Metvix incubation, suggesting enhanced photosensitizer efficacy at this interval. At 48 h, the two-way ANOVA again revealed a significant interaction between K252 and incubation time (*F*(2, 12) = 16.44, *p* = 0.0004; ≈23% variance). The main effects of K252 (*F*(1, 12) = 69.01, *p* < 0.0001; 49% variance) and time (*F*(2, 12) = 14.02, *p* = 0.0007; 20% variance) remained highly significant. The graph (Figure 6e, bottom panel; Supplementary Table S1) shows that K252 consistently potentiated PDT cytotoxicity, though viability differences among incubation times became more apparent, with stronger inhibition maintained at longer times post-PDT.

Altogether, these results demonstrate that K252 pretreatment significantly enhances PDT-induced cytotoxicity in both SCC13 and SCC12 spheroids. In SCC13, the potentiating effect of K252 was strongly time-dependent at both 24 h and 48 h, whereas in SCC12 the interaction was pronounced at 24 h but diminished by 48 h, indicating a cell-line-specific and temporally dynamic response to K252 modulation of PDT efficacy.

Globally, these results suggest that TrkA inhibition may sensitize cSCC cells to oxidative stress induced by PDT, enhancing its therapeutic efficacy and supporting a combinatorial strategy for more effective tumor eradication.

## 3. Discussion

Recent advances in molecular oncology have emphasized the need to identify targetable pathways to improve therapeutic outcomes in advanced cSCC. Among these, the neurotrophic receptor tyrosine kinase (Trk) family emerged as a promising therapeutic target of investigation. cSCC grade of severity involves the progression towards different stages, ranging from the precursor actinic keratosis (AK) to in situ, invasive, and metastatic cSCC. Importantly, the number of people with AK is rapidly growing worldwide, and AK affects up to 60% of the elderly population. Therefore, the public health burden of cSCC is expected to grow. Although most cases are effectively managed with surgical excision, a significant subset presents with aggressive clinical behavior, including perineural invasion, recurrence, and metastatic spread, leading to substantial morbidity and mortality, especially in immunocompromised individuals [8].

Our previous studies have highlighted the key role of the neurotrophin network in maintaining epidermal homeostasis and its significant involvement in skin pathophysiology, particularly in cSCC [13]. Neurotrophin receptors emerged as key regulators of keratinocyte growth and differentiation, responding to both autocrine and paracrine neurotrophin release. In the context of cSCC, we further demonstrated that NTs and their receptors are differentially expressed across disease stages, suggesting a potential association with tumor progression and grade [29]. In the present work, our findings clearly demonstrated that the neurotrophin receptor TrkA plays a pivotal role in cSCC cell survival, growth, and dissemination. Both pharmacological inhibition via K252a and genetic silencing via siRNA significantly impaired cell viability, spheroid development, and metastatic potential, suggesting that the receptor is a functional driver of cSCC malignancy. These data confirm the global effect of K252a on proliferation, viability, and colony formation indicated for other tumor cells, such as the adenocarcinoma of the lung, prostate and stromal tumors of the uterus [55,56,57].

Importantly, our data are in line with other studies that support the feasibility of pharmacologically targeting TrkA using small-molecule inhibitors or monoclonal antibodies [58,59]. Moreover, dual targeting strategies—such as simultaneous inhibition of TrkA and JAK2—have demonstrated synergistic effects in preclinical breast cancer models [23]. Collectively, these works underscore the oncogenic potential of TrkA signaling beyond gene fusions, highlighting its relevance as both a therapeutic target and biomarker in multiple cancers.

The efficacy of PDT depends on multiple factors, such as photosensitizer uptake, tissue oxygenation, and intracellular signaling responses to oxidative stress [53]. However, resistance to PDT or incomplete tumor eradication remains a clinical challenge, particularly in more aggressive or deeply invasive tumors. For this reason, there is growing interest in identifying molecular pathways that modulate the sensitivity of tumor cells to PDT. We demonstrate for the first time that TrkA inhibition can enhance the effectiveness of PDT. This synergistic interaction may be due to the disruption of pro-survival pathways mediated by TrkA signaling, rendering cells more vulnerable to oxidative damage.

On the other hand, by exploiting the zebrafish model, we successfully validated the role of TrkA in promoting metastasis, highlighting its therapeutic potential in advanced cSCC. Currently, patients with advanced cSCC are primarily treated with immunotherapy, specifically the immune checkpoint inhibitors targeting the PD1/PDL1 axis, when surgery or radiotherapy is no longer viable [60,61]. However, a significant number of patients either do not respond to immunotherapies or eventually develop resistance [11]. This underscores the urgent need for additional therapeutic targets to improve treatment outcomes.

Mechanistically, our data suggest that TrkA inhibition disrupts multiple signaling nodes that converge on the AKT and FAK pathways. Both are well-known mediators of survival, migration, and epithelial to mesenchymal transition (EMT) in epithelial cancers. The observed downregulation of phosphorylated AKT and FAK, together with reduced expression of the EMT markers Slug and Snail, indicates that TrkA contributes to sustaining an invasive and mesenchymal-like phenotype in cSCC. This mechanistic link further supports the idea that TrkA acts not merely as a growth regulator but also as a modulator of tumor plasticity and metastatic competence.

Despite the strength of our findings, some limitations should be acknowledged. First, while K252a is a potent inhibitor of TrkA activity, it also targets other kinases at higher concentrations, and therefore genetic validation through TrkA silencing, as performed here, is crucial to confirm specificity. Second, further studies are needed to explore the impact of TrkA inhibition on the tumor microenvironment, immune cell infiltration, and angiogenesis, which are key determinants of cSCC progression in vivo. Future work employing patient-derived xenografts or organotypic skin models may provide additional insights into the clinical relevance of TrkA-targeted therapies.

Overall, our data delineate a model in which TrkA acts as a central regulator of cSCC cell proliferation, survival, and invasion. Its inhibition not only suppresses tumor growth but also sensitizes cells to photodynamic stress, suggesting a dual role as both a molecular driver and promising therapeutic target in cSCC. In conclusion, targeting TrkA may enhance the efficacy of existing drugs when used in combination with current therapies, offering a potential strategy to improve outcomes in aggressive forms of the disease.

## 4. Materials and Methods

### 4.1. Human Keratinocyte Culture

Normal human epidermal keratinocytes (NHEK) were isolated from foreskin surgical samples of healthy skin and cultured as previously described [30]. Tissues were obtained from the Policlinic of Modena, Italy. All subjects gave their informed consent for inclusion before they participated in the study. The study was conducted in accordance with the Declaration of Helsinki, and the protocol was approved by the Ethical Committee of Area Vasta Emilia Nord (Modena, Italy) (Prot. N. 353/2017).

### 4.2. cSCC Cell Line and Spheroids Culture

SCC12B, SCC13 cell lines were purchased from ATCC (Teddington, UK) and maintained in SCC medium (45% DMEM (Dulbecco’s Modified Eagle Medium; Lonza, Basel, Switzerland), 45% Ham’s F12 (Lonza, Basel, Switzerland), 10% Fetal Bovine Serum, 2% L-Glutamine, 1% Penicillin/Streptomycin/Amphotericin, 1M HEPES, and 400 ng/mL Hydrocortisone (Sigma-Aldrich, St. Louis, MO, USA)), as previously described [29]. SCC spheroid formation was obtained by the liquid overlay method, as previously indicated [29].

### 4.3. K252a Treatment and TrkA Silencing

According to experimental requirements, SCC cells and spheroids were treated with the Pan-Trk inhibitor K252a (100, 200 and 400 nM, Sigma-Aldrich, St. Louis, MO, USA), Cisplatin (5 mg/mL, Pfizer, New York, NY, USA), 5-Fluororacil (5-FU; 50 mg/mL), (Accord Healthcare Limited, Middlesex, UK). 24 h after treatment, cells or spheroids were used for the subsequent experiments. For TrkA downregulation, SCC13 and SCC12 cells or spheroids were maintained for 24 h in an antibiotic-free medium and then transfected with TrkA or scrambled siRNA (Dharmacon Inc., Lafayette, CO, USA). 24 h after transfection, cells or spheroids were monitored or collected for subsequent experiments.

### 4.4. Immunofluorescence Staining

NHEK, SCC12, or SCC13 cells, treated as per experimental conditions, were cultured on 8-well chamber slides. At the endpoints, cells were fixed with 4% neutral buffered formalin, and permeabilized with Triton-X100 0.1%. Cells were then incubated with primary antibodies: Ki-67 (1:500; Abcam, Cambridge, UK) or TrkA (1:100; Millipore, Burlington, MA, USA). Cells were labeled with anti-rabbit 546 Alexa Flour secondary antibodies (Invitrogen, Carlsbad, CA, USA). Nuclei were stained with daimidino-2-phenylindole (DAPI; 1:500, Sigma-Aldich, Saint Louis, MO, USA). Samples were analyzed, and images were recorded using a confocal scanning laser microscope (Leica TCS4D, Leica Exton, PA, USA). Quantification of immunofluorescence staining was performed by analyzing six representative fields for each sample by counting the number of positive cells or staining intensity using the ImageJ software version 2.9.0 nCounter plugin (Wayne Rasband, Bethesda, MD, USA) or signal intensity. Scores were made using counting ± SD.

### 4.5. Photodynamic Therapy

Photodynamic therapy (PDT) was performed on SCC12 and SCC13 spheroids by modifying the Austin and Jagdeo 2018 protocol [62], as previously described [29]. Briefly, Spheroids treated with K252a or DMSO (control) were added with aminolevulinic acid (Metvix, 1:10 dilution) for 15 min, 1 or 3 h before exposure to Aktilite CL128 lamp. Immediately after exposure, spheroids underwent a media change.

### 4.6. RT-PCR Western Blotting

The DyNAmo Flash SYBR Green qPCR kit (Thermo Fisher Scientific, Waltham, MA, USA) was used for real-time PCR using an ABI 7500 Real-Time PCR system (Applied Biosystems, Thermo Fisher Scientific, Waltham, MA, USA). The differences in the cycle number past the threshold (DCt) reflect the differences in the initial template concentration in the tested samples. The ΔΔCt method was used to normalize different transcripts (Table 1) and to calculate fold induction relative to the control. The data were analyzed, and samples were quantified using the Sequence Detection Systems software, version 1.2.3, according to the Relative Quantification Study method (Applied Biosystems). Data were normalized to the ACTB housekeeping gene. PCR was carried out at least three times for each sample, and the experiments were performed in triplicate.

NHEK, SCC12, and SCC13 were treated with K252a or DMSO, and SCC12 and SCC13 were transfected with scramble or TrkA siRNA. Total RNA was extracted by PureLink RNeasy Mini Kit (Invitrogen, Carlsbad, CA, USA) as indicated by the manufacturer’s instructions. In total, 1 µg of total RNA was retrotranscribed using the High-Capacity cDNA Reverse Transcription Kit (Applied Biosystems) as described by the manufacturer in a C1000 Touch Thermal Cycler (Bio-Rad Laboratories Inc., Hercules, CA, USA), and cDNA was used for qPCR. Total RNA (500 ng of total RNA extracted was reverse-transcribed, as previously reported [29]. Primer sequences are reported in Table 1.

### 4.7. Western Blotting

Total proteins were run on SDS–PAGE gel, transferred onto a nitrocellulose membrane, and incubated with primary antibodies, listed in Table 2. Membranes were incubated with secondary antibodies, goat anti-mouse or goat anti-rabbit (1:3000; Bio-Rad Laboratories, Hercules, CA, USA). Bands were visualized with a chemiluminescence detection system (Amersham Biosciences UK Limited, Little Chalfont, Buckinghamshire, UK).

### 4.8. MTT Assay

5 × 10^3^ cells from cSCC lines or NEK cells were seeded in 96-well culture plates. For experiments with 3D culture, SCC12 and SCC13 cells were seeded on agar-precoated 96-well culture plates to form spheroids, as previously reportedè [29]. 24, 48 or 120 h after K252a treatment or 48 and 72 h after siRNA transfection, cells or spheroids were incubated with 0.5% MTT (3-(4,5-dimethylthiazol-2-yl)-2,5-diphenyltetrazolium bromide, Sigma-Aldrich). Subsequently, formazan dye crystals were dissolved with 100 µL of Isopropanol for 2D cells and 100 µL of Isopropanol with 0.04 N HCl for spheroids. The MTT reaction was evaluated at 540 nm for cells and with a reference filter of 650 nm for spheroids.

### 4.9. Propidium Iodide Assay

SCC12 and SCC13 spheroids, previously treated with K252a or DMSO or transfected with scramble or TrkA siRNA, as previously mentioned, were washed twice in PBS and incubated for 20′ at RT with a solution of Propidium Iodide (PI, 10 mg/mL; diluted 1:100 in PBS; Sigma-Aldrich, St. Louis, MO, USA). Total area and PI-positive area were calculated by using ImageJ software, as previously reported [24].

### 4.10. Spheroids Invasion Assay

SCC13 cells were seeded on pre-coated 96-well plates with 1.5% of agar to form spheroids. 72 h later after transduction, SCC13 spheroids were implanted in a matrix of human dermal fibroblasts–collagen type I solution composed as follows: DMEM, 200 nM L-glutamine, 10% FBS, 7.5% sodium bicarbonate, 3 mg/mL type I collagen previously extracted from rat tails and 1% penicillin/streptomycin supplemented with 15 × 10^4^/mL of primary human fibroblast derived from skin biopsies. After treatment with K252a (100, 200, and 400 nM), Cisplatin (5 mg/mL), and 5-Fluorouracil (5-FU; 50 mg/mL), according to the experimental conditions, or siRNA transfection, implanted spheroids were monitored and photographed at 24 and 48 h. Invasion area and % of fragmentation were calculated by using ImageJ software, as previously reported [24].

### 4.11. Zebrafish Xenotransplantation

Zebrafish embryos were obtained from natural spawning of the nacre (*mitfa^w2/w2^*) fish line, under local Ethics Committee approval (Aut n. 407/2015-PR). For K252a treatment, normal SCC13 cells or Scramble or TrkA-silenced SCC13 cells, stained with Vybrant Cell-Labeling Solution (Thermo-Fisher Waltham, MA, USA), as previously reported [24], were injected into zebrafish larvae at 2 days post-fertilization (dpf). For K252a treatment, K252a (200 nM) was added directly to the fish water 1 day after SCC13 cell injection (1 dpi; 1-day post-injection). For both experiments, metastases were quantified as previously reported [24]. Imaging was performed by confocal scanning laser microscope (Leica TCS4D, Leica Exton, PA, USA).

### 4.12. Statistical Analysis

A multiparametric *t*-test or two-way ANOVA was performed by GraphPad Prism 9 (we used GraphPad Software, La Jolla, CA, USA, https://www.graphpad.com). Significant *p*-values are indicated with *: 0.01 < *p* < 0.05; **: 0.001 < *p* < 0.01; *** 0.0001 < *p* < 0.001; **** *p* < 0.0001.

## Figures and Tables

**Table 1:** Primer sequence for RT-PCR.

Gene Symbol		Sequences (5′ > 3′)
*β-actin*	FP	TGG ATG ATG ATA TCG CCG CGC TCG
	RP	CAC ATA GGA ATC CTT CTG ACC CA
*BIRC5*	EP	GCA TGG GTG CCC CGA CGT TG
	RP	GCT CCG GCC AGA GGC CTC AA
*NTRK1*	EP	GGC TCC TCG GGA CTG CGA TG
	RP	CAG GAG AGA GAC TCC AGA GCG
*MKI67*	BIORAD, Cat.# qHsaCID0017508	

**Table 2:** Primary antibodies.

Antibody	Provider	Dilution
Mouse anti-human β-actin	Sigma-Aldrich (St. Louis, MO, USA)	1:5000
Rabbit anti-human TrkA	Invitrogen (Carlsbad, CA, USA)	1:1000
Rabbit anti-human Phospho-TrkA	Invitrogen (Carlsbad, CA, USA)	1:1000
Rabbit anti-human Survivin	Cell Signaling Technology (Danvers, MA, USA)	1:1000
Mouse anti-human Cyclin B1	BD Biosciences (Franklin Lakes, NJ, USA)	1:1000
Rabbit anti-human FAK	Cell Signaling Technology (Danvers, MA, USA)	1:1000
Rabbit anti-human Phospho-FAK	Cell Signaling Technology (Danvers, MA, USA)	1:1000
Anti-human AKT	Cell Signaling Technology (Danvers, MA, USA)	1:1000
Anti-human phosphor-AKT	Cell Signaling Technology (Danvers, MA, USA)	1:1000
Anti-human PCNA	Abcam (Cambridge, UK)	1:1000
Rabbit anti-human KRT13	Abcam (Cambridge, UK)	1:1000
Rabbit anti-human Slug	Cell Signaling Technology (Danvers, MA, USA)	1:1000
Rabbit anti-human Snail	Cell Signaling Technology (Danvers, MA, USA)	1:1000

## Data Availability

The original contributions presented in this study are included in the article and Supplementary Materials. Further inquiries can be directed to the corresponding author.

## Supplementary Materials

The following supporting information can be downloaded at https://www.mdpi.com/article/10.3390/ijms262110434/s1.
