# Supplementary material for: Trk Signaling Inhibition Reduces cSCC Growth and Invasion in In Vitro and Zebrafish Models and Enhances Photodynamic Therapy Outcome
Source: Int J Mol Sci. 2025 Oct 27;26(21):10434. doi: 10.3390/ijms262110434 (PMC12610016; doi:10.3390/ijms262110434)
Supplement: Supplementary file 1 [file ijms-26-10434-s001.zip › Supplementary Figures S1-S3.pdf]

Supplementary Information

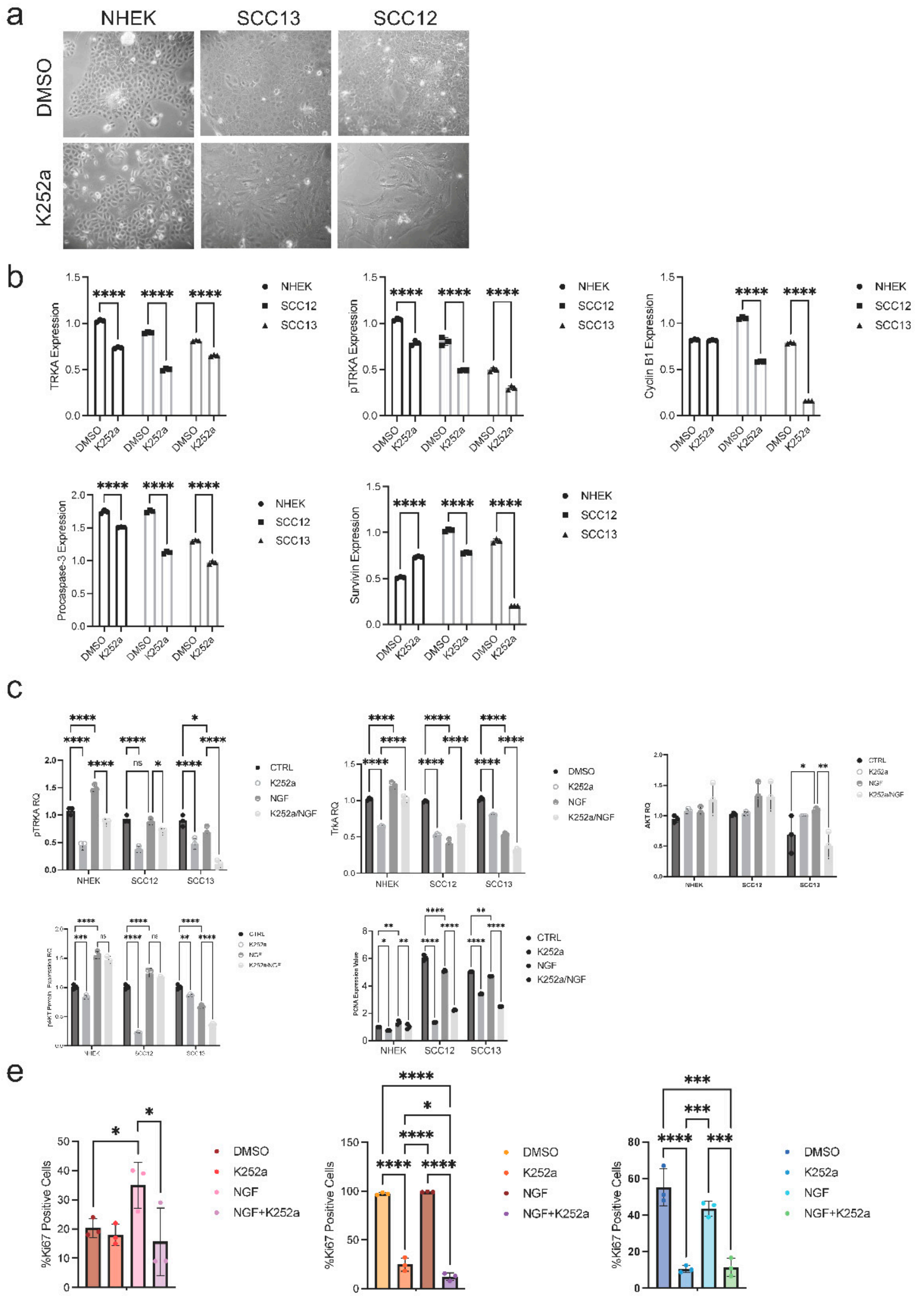

Supplementary Figure S1: K252a treatment affects cSCC growth and viability. a) Representative image of NHEK, SCC12 and SCC13 cells treated with K252a (200 nM) or DMSO (Control) at 24 hours. b) Quantification of TrkA, P-TrkA, Cyclin B1, Survivin, and Pro-caspase-3 expression evaluated at 24 hrs after K252a (200 nM) treatment by ImageJ software c) Densitometry analysis of TrkA, P-TrkA, pAKT, AKT and PCNA expression evaluated at 24 hrs after K252a (200 nM) and or NGF (100 ng/ml) treatment by ImageJ software. d) Quantification of Ki67 expression by ImageJ software. For all experiments, statistical analysis was performed using two-way ANOVA. The results are represented as mean ± SD. p-values are indicated as the following *: 0.01<p<0.05; 0.001<p<0.01; ***0.0001<p<0.001; **** p<0.0001.

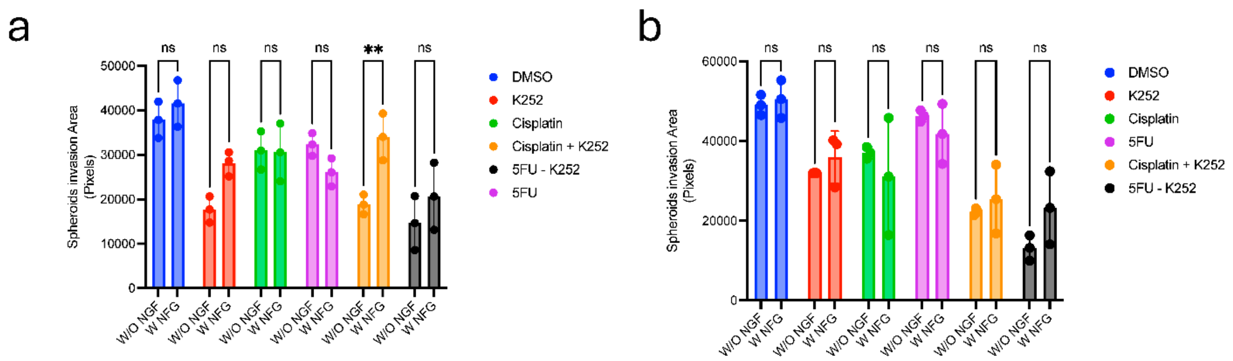

Supplementary Figure S2: Spheroid invasion modulation by K252a and/or chemotherapeutics is not influenced by the presence of NGF. Total invasion area at 24 h (a) or 48 h (b) measured by ImageJ software. Statistical analysis was performed using two-way ANOVA. The results are represented as mean ± SD. p-values are indicated as the following: ns=not significant, **0.001<p<0.01.

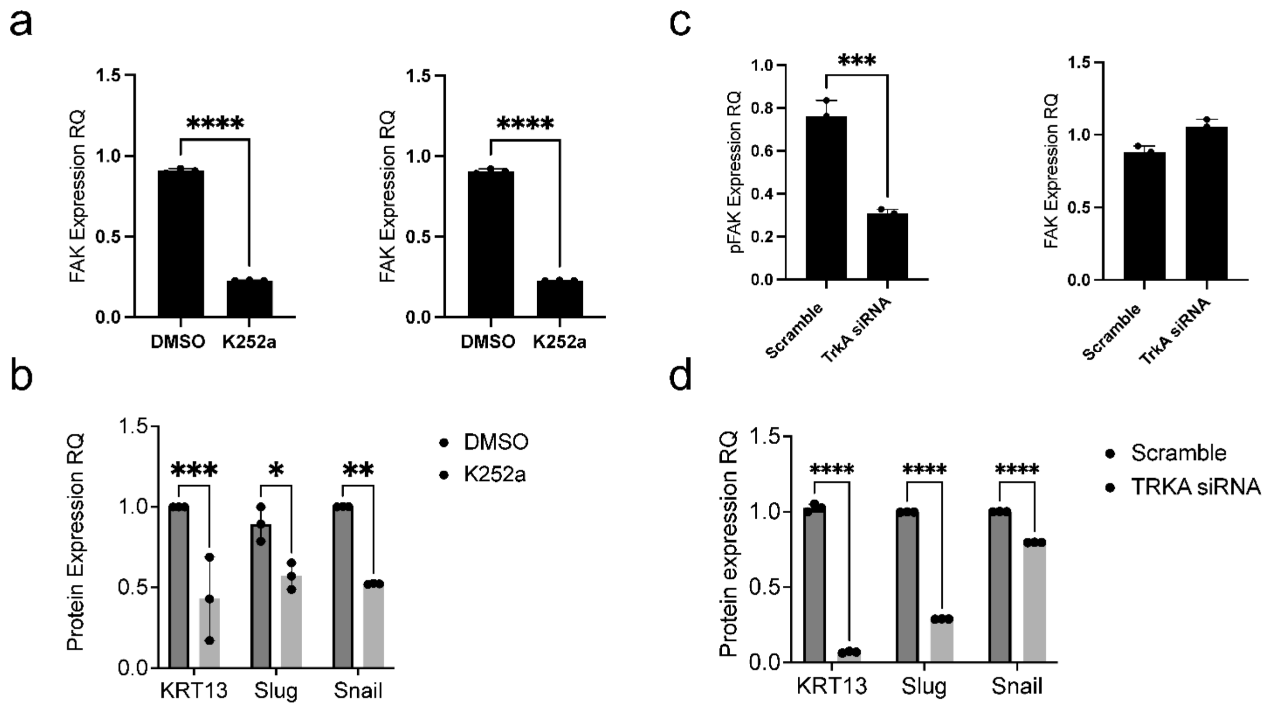

Supplementary Figure S3. Densitometry of cSCC cells treated with K252a or transfected with siRNAs. For all the indicated proteins, ImageJ software analysis was used to evaluate band intensity; β-actin was used as loading control. Statistical analysis was performed using two-way ANOVA. (a) and (c) showed the quantification of FAK and phospho-FAK (pFAK) expression in K252a or TrkA siRNA treated cells vs their control (DMSO or Scramble, respectively). (b) and (d) showed the quantification of KRT13, Slug and Snail expression in K252a or TrkA siRNA treated cells vs their control (DMSO or Scramble, respectively). The results are represented as mean ± SD. p-values are indicated as the following *: 0.01<p<0.05; ** 0.001<p<0.01; ***0.0001<p<0.001; **** p<0.0001.
