# Supplementary material for: Trk Signaling Inhibition Reduces cSCC Growth and Invasion in In Vitro and Zebrafish Models and Enhances Photodynamic Therapy Outcome
Source: Int J Mol Sci. 2025 Oct 27;26(21):10434. doi: 10.3390/ijms262110434 (PMC12610016; doi:10.3390/ijms262110434)
Supplement: Supplementary file 1 [file ijms-26-10434-s001.zip › Supplementary Table S1.pdf]

Data analyzed: SCC12 24 hrs

Source of Variation	Degrees of Freedom	Sum of Squares	Mean square
Column Factor	1	0.4687	0.4687
Row Factor	2	0.3939	0.1970
Interaction	2	0.1616	0.08079
Residual (error)	12	0.07924	0.006603
Total	17	1.103	

Does Column Factor have the same effect at all values of Row Factor?
Interaction accounts for 14.64% of the total variance.
F = 12.24. DFn = 2, DFd = 12
The P value = 0.0013
If there is no interaction overall, there is a 0.13% chance of randomly observing so much interaction in an experiment of this size. The interaction is considered very significant.

Since the interaction is statistically significant, the P values that follow for the row and column effects are difficult to interpret.

Does Column Factor affect the result?
Column Factor accounts for 42.47% of the total variance.
F = 70.98. DFn = 1, DFd = 12
The P value is < 0.0001
If Column Factor has no effect overall, there is a less than 0.01% chance of randomly observing an effect this big (or bigger) in an experiment of this size. The effect is considered extremely significant.

Does Row Factor affect the result?
Row Factor accounts for 35.7% of the total variance.
F = 29.83. DFn = 2, DFd = 12
The P value is < 0.0001
If Row Factor has no effect overall, there is a less than 0.01% chance of randomly observing an effect this big (or bigger) in an experiment of this size. The effect is considered extremely significant.

Data analyzed: SCC12 48 hrs

Source of Variation	Degrees of Freedom	Sum of Squares	Mean square
Column Factor	1	0.006062	0.006062
Row Factor	2	0.001640	0.0008200
Interaction	2	0.0004696	0.0002348
Residual (error)	12	0.001018	8.480e-005
Total	17	0.009190	

Does Column Factor have the same effect at all values of Row Factor?
Interaction accounts for 5.11% of the total variance.
F = 2.77. DFn = 2, DFd = 12
The P value = 0.1026
If there is no interaction overall, there is a 10% chance of randomly observing so much interaction in an experiment of this size. The interaction is considered not significant.

Does Column Factor affect the result?
Column Factor accounts for 65.97% of the total variance.
F = 71.49. DFn = 1, DFd = 12
The P value is < 0.0001
If Column Factor has no effect overall, there is a less than 0.01% chance of randomly observing an effect this big (or bigger) in an experiment of this size. The effect is considered extremely significant.

Does Row Factor affect the result?
Row Factor accounts for 17.85% of the total variance.
F = 9.67. DFn = 2, DFd = 12
The P value = 0.0032
If Row Factor has no effect overall, there is a 0.32% chance of randomly observing an effect this big (or bigger) in an experiment of this size. The effect is considered very significant.

Data analyzed: SCC13 24 hrs

Source of Variation	Degrees of Freedom	Sum of Squares	Mean square
Column Factor	1	1.479	1.479
Row Factor	2	0.06510	0.03255
Interaction	2	0.5077	0.2539
Residual (error)	12	0.05843	0.004869
Total	17	2.110	

Does Column Factor have the same effect at all values of Row Factor?
Interaction accounts for 24.06% of the total variance.
F = 52.14. DFn = 2, DFd = 12
The P value is < 0.0001
If there is no interaction overall, there is a less than 0.01% chance of randomly observing so much interaction in an experiment of this size. The interaction is considered extremely significant.

Since the interaction is statistically significant, the P values that follow for the row and column effects are difficult to interpret.

Does Column Factor affect the result?
Column Factor accounts for 70.09% of the total variance.
F = 303.80. DFn = 1, DFd = 12
The P value is < 0.0001
If Column Factor has no effect overall, there is a less than 0.01% chance of randomly observing an effect this big (or bigger) in an experiment of this size. The effect is considered extremely significant.

Does Row Factor affect the result?
Row Factor accounts for 3.085% of the total variance.
F = 6.69. DFn = 2, DFd = 12
The P value = 0.0112
If Row Factor has no effect overall, there is a 1.1% chance of randomly observing an effect this big (or bigger) in an experiment of this size. The effect is considered significant.

Data analyzed: SCC13 48 hrs

Source of Variation	Degrees of Freedom	Sum of Squares	Mean square
Column Factor	1	0.05120	0.05120
Row Factor	2	0.02080	0.01040
Interaction	2	0.02440	0.01220
Residual (error)	12	0.008903	0.0007419
Total	17	0.1053	

Does Column Factor have the same effect at all values of Row Factor?
Interaction accounts for 23.17% of the total variance.
F = 16.44. DFn = 2, DFd = 12
The P value = 0.0004
If there is no interaction overall, there is a 0.037% chance of randomly observing so much interaction in an experiment of this size. The interaction is considered extremely significant.

Since the interaction is statistically significant, the P values that follow for the row and column effects are difficult to interpret.

Does Column Factor affect the result?
Column Factor accounts for 48.62% of the total variance.
F = 69.01. DFn = 1, DFd = 12
The P value is < 0.0001
If Column Factor has no effect overall, there is a less than 0.01% chance of randomly observing an effect this big (or bigger) in an experiment of this size. The effect is considered extremely significant.

Does Row Factor affect the result?
Row Factor accounts for 19.75% of the total variance.
F = 14.02. DFn = 2, DFd = 12
The P value = 0.0007
If Row Factor has no effect overall, there is a 0.073% chance of randomly observing an effect this big (or bigger) in an experiment of this size. The effect is considered extremely significant.
